# The Impact of Humble Leadership on the Green Innovation Performance of Chinese Manufacturing Enterprises: A Moderated Mediation Model

**DOI:** 10.3390/bs15091170

**Published:** 2025-08-28

**Authors:** Tianye Tu, MyeongCheol Choi

**Affiliations:** Department of Business Management, Gachon University, Seongnam 13120, Republic of Korea; tutianye2018@yeah.net

**Keywords:** humble leadership, caring ethical climate, green innovation performance, organizational structure, organizational resource slack

## Abstract

Currently, environmental issues negatively affect both firm performance and economic development, prompting society to expect enterprises to address these issues more effectively. In response, organizations, particularly manufacturing enterprises, have begun to adopt green innovation. This study examines how humble leadership in enterprise management affects organizational green innovation performance. Additionally, this study explores the mediating role of the organizational caring ethical climate and the moderating roles of the organizational structure and unabsorbed organizational resource slack. This study involved top managers from 357 manufacturing enterprises in Zhejiang Province, yielding 306 valid questionnaires. The Hierarchical regression technique is used to analyze the survey data. An analysis of the data shows that humble leadership positively affects organizational green innovation performance, with the organizational caring ethical climate serving as a mediator. Furthermore, the organizational structure and organizational resource slack positively moderate the effect of the organizational caring ethical climate on green innovation performance. This study validates and enriches social learning theory; social exchange theory; conservation of resource theory; and ability, motivation, and opportunity theory. It also provides new insights into the relationship between humble leadership and green innovation performance and expands research on the moderators of the relationship between the organizational caring ethical climate and green innovation performance. The findings suggest that managers of manufacturing enterprises should adopt humble leadership, promote a caring ethical climate, and enhance cooperation with stakeholders.

## 1. Introduction

Currently, environmental problems significantly threaten both firm performance and economic development (Huang et al., 2016; Tseng et al., 2013; Song et al., 2020; Al Koliby et al., 2025). In response, environmental protection regulations have imposed higher requirements on corporate environmental responsibilities and consumer environmentalism has grown in popularity (Song et al., 2020; Al Koliby et al., 2025; Albort-Morant et al., 2018). In response to these pressures, organizations, particularly manufacturing enterprises, use green innovation as a key strategy to promote their sustainable development (Song et al., 2020). Green innovation involves technological advancements that save energy, decrease environmental pollution, recycle resources, and manage environmental protection (Y. S. Chen et al., 2006). In addition to environmental benefits, this strategy can lead to many other positive outcomes for organizations. Organizations that embrace green innovation can seize new market opportunities, enhance existing customers’ loyalty, and attract potential customers (Sun et al., 2021). Consequently, green innovation can help organizations achieve a unique competitive advantage (Sun et al., 2021). Therefore, improving the green innovation performance of manufacturing enterprises has become a focal point for both scholars and practitioners.

Many scholars consider leadership as a crucial predictor of the green innovation performance in organizations (Begum et al., 2022; Akhtar et al., 2023; Arici & Uysal, 2022). For example, Mendez-Picazo et al. (2024) find that digital transformation is beneficial to the green innovation performance. There is increasing interest in how leadership styles affect organizational green innovation performance. Begum et al. (2022) demonstrate that green transformation leadership positively affects this performance. Similarly, studies have shown a positive relationship between responsible leadership and the green innovation performance (Akhtar et al., 2023). Additionally, X. Su et al. (2020) find that environmental leadership positively correlates with the green innovation performance in organizations. However, the effect of humble leadership on the organizational green innovation performance remains underexplored. Humble leadership refers to a series of leader behaviors such as accurately assessing oneself, valuing others’ contributions and strengths, and being open to feedback or new ideas (Owens & Hekman, 2012). While Sun et al. (2021) examine the relationship between CEO humility and corporate green innovation, the influence of humble leadership within corporate management on the green innovation performance is still unclear. Notably, the (group) leadership style at the management level differs from that at the individual CEO level. Thus, this study aims to explore the relationship between humble leadership in the management of manufacturing enterprises and their green innovation performance. Compared with the studies conducted by Begum et al. (2022), X. Su et al. (2020), and Akhtar et al. (2023), the present research investigates the impact of a novel form of leadership—humble leadership—on organizational green innovation performance, thereby enriching the literature on the antecedents of green innovation performance. While Sun et al. (2021) focused on the influence of individual leadership on green innovation performance, this study extends the scope by examining the impact of collective leadership at the group level, contributing to a deeper understanding of green innovation performance from a group-level perspective.

This study examines the mediating role of an organizational caring ethical climate in the relationship between humble leadership and the organizational green innovation performance. Sun et al. (2021) investigate the mediating role of a green business strategy between CEO humility and the corporate green innovation performance, noting that scholars have a limited understanding of how humble leadership affects the corporate green innovation performance. Thus, exploring the mediating role of organizational caring ethical climate effectively addresses this research gap. Additionally, humble leadership positively affects various psychological variables, such as altruism and organizational psychological safety (Kelemen et al., 2023). This supports the inference that humble leadership can enhance positive organizational climates, such as a caring ethical climate. Furthermore, research indicates that a positive organizational climate can enhance innovation and performance (Kwon Choi et al., 2013). Therefore, a caring ethical climate is expected to positively affect the organizational green innovation performance and is proposed to mediate the relationship between humble leadership and green innovation performance. Compared with the study by Sun et al. (2021), the present research introduces a novel mediating variable—a caring ethical climate—into the relationship between humble leadership and the organizational green innovation performance. This contributes to the existing literature by enriching the understanding of the psychological mechanisms through which humble leadership influences the green innovation performance. Furthermore, although some existing studies have provided indirect evidence regarding the mediating role of a caring ethical climate (Kelemen et al., 2023; Kwon Choi et al., 2013; Andersson et al., 2020), they lack direct theoretical development and empirical validation. This study addresses this gap by offering both theoretical grounding and empirical support.

Furthermore, this study explores the moderating role of the organizational structure in the relationship between a caring ethical climate and green innovation performance. Some studies have focused on the moderator of the relationship between an ethical climate and innovation. For example, Kwon Choi et al. (2013) examine the moderating role of support for innovation and performance evaluation in the relationship between an ethical climate and organization innovation, and Santiago-Torner (2023) studies the moderating role of work autonomy in the relationship between an ethical climate and employee creativity. However, there is a notable lack of research on how the organizational structure might affect the relationship between the ethical climate and organizational green innovation performance. This research gap highlights the significance of this study’s objective. Meanwhile, substantial evidence supports the link between the organizational structure and innovation performance, with scholars agreeing that an organic organizational structure promotes innovation (Pierce & Delbecq, 1977; Polansky & Hughes, 1986; Dedahanov et al., 2017). Therefore, it is reasonable to propose that, when an organizational structure is closer to an organic model, a caring ethical climate is more likely to enhance the organizational green innovation performance. Although extensive evidence has demonstrated that organic organizational structures facilitate organizational innovation (Pierce & Delbecq, 1977; Polansky & Hughes, 1986; Dedahanov et al., 2017), it remains unclear whether an organizational structure moderates the relationship between humble leadership and the organizational innovation performance. It is important to note that the preceding discussion has identified two distinct mechanisms: the direct effect of a single variable on another, and the interactive effect of two variables on a third. This study contributes to the literature by advancing the understanding of the moderating mechanism and the interaction effect between humble leadership and organizational structure on innovation outcomes.

This study also examines how unabsorbed organizational resource slack moderates the relationship between an organization’s caring ethical climate and its green innovation performance. Previous research has primarily focused on the moderating roles of support for innovation, performance evaluation, and work autonomy in the relationship between an ethical climate and innovation (Kwon Choi et al., 2013; Santiago-Torner, 2023). However, scarce research has addressed the moderating effects of organizational resource slack in this context. In contrast, substantial evidence suggests that unabsorbed organizational resource slack benefits organizational innovation (Tan & Peng, 2003; Marlin & Geiger, 2015). Therefore, it is highly likely that unabsorbed organizational resource slack significantly affects the relationship between a caring ethical climate and the organizational green innovation performance.

In the final section of the introduction, the authors summarize the objectives of this study and provide an overview of this paper’s structure. This research pursues four primary objectives. First, it seeks to investigate the relationship between humble leadership in manufacturing enterprises and their green innovation performance. Second, it aims to examine the mediating role of a caring ethical climate in the relationship between humble leadership and the organizational green innovation performance. Third, this study explores the moderating effect of the organizational structure on the relationship between a caring ethical climate and the green innovation performance. Finally, it assesses how unabsorbed organizational resource slack moderates the relationship between a caring ethical climate and the green innovation performance. Regarding the structure of this paper, the first section presents the introduction, which outlines the social context, identifies the research gap, and states this study’s objectives. This is followed by the theoretical foundation, which discusses the key theories underpinning the research. The subsequent section provides a comprehensive literature review and the development of hypotheses, drawing on relevant theoretical frameworks. Next, the research methodology section describes the data collection and analysis procedures employed in this study. This study analyzed 306 valid questionnaires from top managers from manufacturing enterprises in Zhejiang province. The results section then presents the empirical findings. The results support the mediating role of organizational caring ethical climates and the moderating role of the resource slack and organizational structure in the relationship between humble leadership and the organizational green innovation performance. Finally, this paper concludes with a discussion of the theoretical and practical implications of the research.

## 2. Literature Review and Hypotheses Development

### 2.1. Theoretical Foundation

Social learning theory posits that learning occurs through social interactions (Bandura & Walters, 1977). Specifically, individuals identify certain people as role models and acquire knowledge by observing, learning from, and imitating their behaviors (Bandura & Walters, 1977). In the workplace, employees naturally regard their leaders as role models and tend to mimic their actions. Humble leaders typically demonstrate behaviors such as acknowledging others’ strengths and contributions and being open to others’ opinions (Owens & Hekman, 2012). According to social learning theory, subordinates under the influence of humble leadership are likely to exhibit similar behaviors. These behaviors are closely associated with a caring ethical climate within the organization. Therefore, this study adopts social learning theory to explain the relationship between humble leadership and a caring organizational ethical climate.

According to social exchange theory, individuals are inclined to reciprocate the efforts and support they receive from others, and they also expect their own contributions to be acknowledged in return (Myers et al., 2020). This theory has been widely validated in organizational research. Scholars have found that when employees perceive goodwill from their organization, they tend to respond with greater work effort and innovative behavior (Alalsheikh et al., 2022). Since a caring ethical climate is closely related to employees’ perception of organizational goodwill, this study applies social exchange theory to explain how a caring organizational ethical climate promotes the green innovation performance.

Conservation of Resources (COR) theory is one of the most influential theories in the field of organizational behavior. It was first proposed by psychologist Hobfoll in 1989, originally focusing on the domain of stress (Hobfoll, 1989). The core premise of the theory is that individuals experience a sense of threat when they face actual or a potential loss of valuable resources. To cope with this threat, they strive to acquire, conserve, and maintain resources that are important to them. One key proposition of COR theory is the “primacy of resource gain” effect, which suggests that individuals with greater initial resources are better able to avoid resource loss, more willing to invest in resource-generating activities, and thus more likely to experience a resource gain spiral (Hobfoll et al., 2018). Employees’ innovative behavior is considered a form of resource investment, as it involves taking risks and expending effort with the aim of generating innovation outcomes and achieving greater returns. A caring ethical climate in organizations provides employees with various psychological resources. Therefore, this study applies COR theory to explain the positive impact of a caring organizational ethical climate on green innovation performance.

The AMO theory, which is widely recognized as a foundational framework for understanding the determinants of performance, posits that performance is driven by three key components: Ability, Motivation, and Opportunity (Armstrong & Baron, 2005). These elements are not only essential individually, but also mutually reinforcing in enhancing performance (Armstrong & Baron, 2005). Existing research has shown that the organizational structure and unabsorbed resource slack can influence a firm’s innovation capability (Dedahanov et al., 2017; Vanacker et al., 2017). As discussed earlier, both Conservation of Resources theory and Social Exchange theory suggest that a caring organizational ethical climate enhances employees’ motivation to innovate. Therefore, this study applies the AMO theory to explain the moderating roles of the organizational structure and unabsorbed resource slack in the positive relationship between a caring organizational ethical climate and green innovation performance.

### 2.2. Humble Leadership and Green Innovation Performance

Arrogant and narcissistic leaders often cause business mistakes and accounting scandals (Kelemen et al., 2023). Consequently, scholars and practitioners place high importance on humble leadership (Kelemen et al., 2023). Humble leadership has been found to have a positive impact on individual job satisfaction and performance (Kelemen et al., 2023). Moreover, some studies also validate the positive relationship between humble leadership and organizational performance and innovation (Kelemen et al., 2023). Many researchers have examined this concept. Owens and Hekman (2012) conducted in-depth interviews to identify a set of behavior of humble leaders that include an accurate self-assessment, appreciation of others’ contributions and strengths, and openness to feedback or new ideas. Furthermore, they also find that humble leadership can lead to positive follower behavior such as engagement. Ou et al. (2014) view it as a self-recognition that something is superior to oneself, leading to low self-focus, self-awareness, a pursuit of self-excellence, openness to feedback, and admiration of others. Liu (2016) defines it as recognizing one’s weaknesses and disadvantages, admiring followers’ strengths and contributions, and being receptive to new information. The three definitions differ in emphasis: Owens and Hekman (2012) focus on behavioral expressions, Ou et al. (2014) emphasize internal self-perception and low self-focus, while Liu (2016) highlights relational humility toward followers. Owens and Hekman’s (2012) framework is the most influential in this field with over three-quarters of the research that measures humble leadership relying on this framework. The scale developed by Owens and Hekman (2012) was constructed in accordance with established guidelines for scale development, ensuring its methodological rigor. Moreover, subsequent research by Owens et al. (2013) has provided evidence of the scale’s reliability across multiple languages (e.g., Chinese, Korean, Portuguese, and English) and diverse national contexts, including the United States, China, Singapore, South Korea, Pakistan, India, and Vietnam. Therefore, the author adopts the definition of humble leadership proposed by Owens and Hekman (2012).

As environmental issues gain importance, enterprises must focus more on environmental protection, making green innovation essential to their strategies. Green innovation aids in value creation, a competitive advantage, and performance enhancement for businesses (Albort-Morant et al., 2018). Similar concepts, such as environmental and ecological innovation, also emphasize reducing negative effects on the environment (Arici & Uysal, 2022). Green innovation incorporates both green process innovation and green product innovation, including technological innovations that save energy, reduce environmental pollution, recycle resources, and manage environmental protection (Y. S. Chen et al., 2006).

Scholars have discovered that humble leadership positively affects organizational innovation performance and corporate social responsibility (Kelemen et al., 2023). As green innovation closely relates to corporate social responsibility, it is reasonable that humble leadership positively influences an organization’s green innovation performance (Kelemen et al., 2023). Furthermore, Sun et al. (2021) found that a CEO’s humble leadership positively affects the corporate green innovation performance, because such leaders are more likely to perceive the environmental requirements of stakeholders as important, which encourages them to adopt green business strategies. These strategies positively affect corporate green innovation. As the CEO is a core member of the top management team, and given that the humble leadership of the CEO has a positive impact on the organizational green innovation performance, it is likely that the humble leadership of the entire top management team may also exert a positive influence on the green innovation performance. Notably, while Sun et al. (2021) investigated the effect of CEO humility on corporate green innovation, their study was limited to individual-level leadership (i.e., CEO). In contrast, our study examines humble leadership as a collective characteristic of the entire management team. This approach provides a broader organizational perspective, capturing how humble leadership at the top management team level can influence the green innovation performance across the enterprise. Thus, our study both extends and differentiates from prior research by focusing on the group-level leadership dynamic.

According to social learning theory (Bandura & Walters, 1977), individuals model behaviors after credible role models within their environment. In organizational settings, humble leaders serve as such role models, demonstrating open-mindedness, humility, and appreciation for others. When top management exhibits humble leadership, these behaviors are likely to permeate the organization, fostering an environment supportive of innovation and adaptive change, including green innovation initiatives.

**H1.**  *The humble leadership of enterprise management positively affects organizational green innovation performance.*

### 2.3. Humble Leadership and an Organizational Caring Ethical Climate

Recently, there has been a growing interest in the organizational ethical climate because it significantly affects employees’ behavior (Koskenvuori et al., 2019; Z. Chen & Tang, 2024). An organizational ethical climate refers to the shared perceptions of ethical standards that define ethically correct behaviors and how an organization handles ethical issues (Bart & Cullen, 1987). They also categorize an organizational ethical climate into five types: independence, caring, rules, law and code, and instrumental. A caring climate is one in which consideration for others is fundamental. In this type of climate, employees care about the benefits of others both inside and outside the organization (Dinc & Huric, 2017).

An organizational caring ethical climate has a compact relationship with humble leadership. Here is the mechanism for the relationship. According to social learning theory, individuals observe and mimic behaviors from role models (Bandura & Walters, 1977). In the workplace, leaders serve as significant role models for their followers. Consequently, followers naturally adopt behaviors exhibited by humble leaders, such as valuing others’ contributions and being open to their advice. These behaviors positively affect the organizational performance (Kelemen et al., 2023) and demonstrate a high regard for the organization. Humble leadership also promotes a caring ethical climate and has been shown to positively affect altruism (Kelemen et al., 2023), which is a spontaneous helping behavior. As altruism is often a result of a caring ethical climate, humble leadership is likely to positively affect this climate, yielding the following hypothesis:

**H2.**  *Humble leadership positively affects the organizational caring ethical climate.*

### 2.4. Mediating Role of a Caring Ethical Climate

Research indicates that a caring ethical climate can enhance employees’ job satisfaction and organizational commitment (Koskenvuori et al., 2019), thus encouraging them to exert more effort in improving the organizational performance. As green innovation performance is a form of organizational performance, it is highly likely that a caring ethical climate can also improve an organization’s green innovation performance.

In a caring ethical climate, organizations demonstrate concern for their members, who, in turn, care for each other (Z. Chen & Tang, 2024). Social exchange theory (Blau, 1964) posits that when employees perceive benevolence and support from their organization, they are likely to reciprocate through positive behaviors, such as increased effort and innovation. In a caring ethical climate, employees feel valued and respected, strengthening their commitment to organizational goals—including green innovation. The conservation of resources (COR) theory further suggests that a supportive organizational climate provides psychological and social resources, which reduce risk aversion and enable employees to invest in creative activities (Hobfoll et al., 2018). Empirical studies confirm that ethical and caring climates promote a higher level of innovation and performance (Kwon Choi et al., 2013; Andersson et al., 2020). Therefore, a caring ethical climate is expected to mediate the effect of humble leadership on green innovation performance.

Additionally, a caring ethical climate promotes mutual trust and assistance among organizational members (Z. Chen & Tang, 2024), providing them with essential psychological and social resources. According to the conservation of resources (COR) theory, abundant initial resources encourage employees to engage in resource investment behaviors to secure additional benefits (Spanouli & Hofmans, 2021). Innovation—characterized by a high risk and return—is a typical example of such a behavior. Consequently, a caring ethical climate motivates employees to invest more effort in innovation, which enhances the organization’s green innovation performance. In summary, prior paragraphs show that humble leadership promotes an organizational caring ethical climate that can lead to a better organizational green innovation performance. This leads to the following hypothesis:

**H3.**  *A caring ethical climate mediates the relationship between the humble leadership of enterprise management and organizational green innovation performance.*

### 2.5. Moderating Role of Organizational Structure

The organizational structure defines the formal system of roles and administrative mechanisms that manage resource flows and work activities (Olson et al., 1995). Two classic types of organizational structures are the organic and mechanistic models (Z. Su et al., 2011; Kessler et al., 2017). The mechanistic organizational structure features a high emphasis on formally defined rules, centralized decision making, and tight control over information (Z. Su et al., 2011; Kessler et al., 2017). In contrast, the organic organizational structure is characterized by fewer formal rules and regulations, the low centralization of authority, and open communication (Z. Su et al., 2011; Kessler et al., 2017). It has been found that an organic organizational structure can improve the corporate social responsibility performance (Pasricha et al., 2018). Moreover, an organic organizational structure also positively affects the intellectual capital of enterprises (Ramezan, 2011).

An organic organizational structure promotes organizational innovation (Dedahanov et al., 2017; Kessler et al., 2017). Dedahanov et al. (2017) explain that the key features of this structure—low centralization and low formalization—positively affect innovation. Low centralization allows more members to participate in the decision-making process, enhancing the diversity and exchange of ideas, which are crucial for innovation. Additionally, low formalization enables employees to communicate and explore new ideas freely, further supporting innovation within the organization.

According to the ability, motivation, and opportunity (AMO) theory, performance depends on three elements—ability, motivation, and opportunity—which complement each other (Armstrong & Baron, 2005). Therefore, it is reasonable to infer that an organization’s green innovation performance also depends on these factors. A caring ethical climate can enhance organizational members’ motivation to innovate. Additionally, an organic organizational structure can encourage the diversity and exchange of ideas (Dedahanov et al., 2017), thereby enhancing members’ ability to innovate. Consequently, the organizational structure and a caring ethical climate work together to promote organizational green innovation.

**H4.**  *The organizational structure moderates the relationship between an organizational caring ethical climate and organizational green innovation performance.*

**H4a.**  *When the organizational structure is more organic, the positive relationship between a caring ethical climate and organizational green innovation performance becomes stronger.*

**H4b.**  *When the organizational structure is more mechanistic, the positive relationship between a caring ethical climate and organizational green innovation performance becomes weaker.*

### 2.6. Moderating Role of Organizational Resource Slack

Organizational resource slack refers to a group of resources that exceed the minimum necessary to maintain normal operations within an organization (Vanacker et al., 2017). There are two types of resource slack: absorbed and unabsorbed (Marlin & Geiger, 2015). Unabsorbed resource slack refers to resources that are not currently engaged in operations and are readily accessible for redeployment within the organization (Marlin & Geiger, 2015; Vanacker et al., 2017). In contrast, absorbed resources are already involved in current operations and are difficult to recover or redeploy (Marlin & Geiger, 2015; Vanacker et al., 2017). This study focuses on unabsorbed resource slack, because these resources are easier to deploy for innovation. It has been found that absorbed resource slack and unabsorbed resource slack both positively influence the performance of small businesses (Jifri et al., 2016). Moreover, unabsorbed slack exerts a more positive impact on a firm’s future profitability compared to absorbed slack (Argiles-Bosch et al., 2016).

Unabsorbed resource slack positively affects the firm’s performance (Tan & Peng, 2003; Marlin & Geiger, 2015) and innovation (Vanacker et al., 2017). Specifically, unabsorbed resource slack enables organizations to overcome the fear of failure and conduct more innovation experiments (Vanacker et al., 2017). Consequently, it is reasonable that unabsorbed resource slack positively affects green innovation performance.

**Figure 1:**
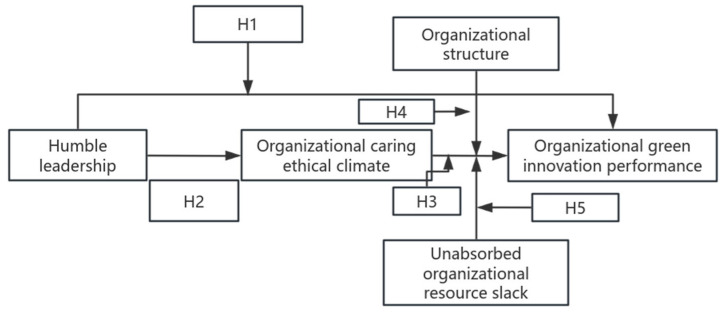
The proposed model.

According to AMO theory, the ability, motivation, and opportunity factors complement each other in stimulating performance (Armstrong & Baron, 2005). As previously mentioned, a caring ethical climate can motivate green innovation. Additionally, unabsorbed resource slack provides organizations with the opportunity to pursue green innovation. Therefore, these two factors supplement each other in determining the organizational green innovation performance. This paper shows the complete proposed model by Figure 1.

**H5.**  *Organizational resource slack moderates the relationship between an organizational caring ethical climate and organizational green innovation performance.*

**H5a.**  *When organizational unabsorbed resource slack is high, the positive relationship between a caring ethical climate and organizational green innovation performance becomes stronger.*

**H5b.**  *When organizational unabsorbed resource slack is low, the positive relationship between a caring ethical climate and organizational green innovation performance becomes weaker.*

## 3. Research Method

### 3.1. Sample and Procedure

With the support of local chambers of commerce, the authors sent e-mails to 650 manufacturing enterprises in Zhejiang province, inviting them to participate in this study by explaining its aim, significance, content, and process. In the e-mails, the authors requested that enterprises willing to participate recommend a member of their top management team who was knowledgeable about the enterprise’s leadership, climate, and green innovation to complete the questionnaire. Two weeks later, 296 manufacturing enterprises accepted the invitation and provided recommendations, 156 rejected it, and 198 did not respond. To increase the sample size, the authors sent follow-up invitations to the 198 non-responding enterprises. After another two weeks, 61 of these enterprises accepted the invitation and made their recommendations. Consequently, 357 manufacturing enterprises finally agreed to participate in this study.

After successfully inviting the enterprises, the authors conducted a survey by sending questionnaire e-mails to the 357 respondents recommended by these enterprises. Two weeks later, 346 respondents replied. Upon reviewing the completeness and response patterns of the filled questionnaires, the authors excluded 40 invalid questionnaires, resulting in 306 valid ones. The valid response rate among the invited enterprises was 85.7%. Inviting top managers to represent their enterprises in the survey offers several benefits. First, top managers typically possess strong conceptual skills, which help them understand and accurately answer the survey questions, thereby improving the reliability and validity of the data. Additionally, their familiarity with the overall operations of the enterprise enables them to provide precise responses.

Among the 306 enterprises analyzed, 8.2% (25 enterprises) employ fewer than 100 individuals, 47.4% (145 enterprises) employ 100–250 individuals, 37.6% (115 enterprises) employ 250–500 individuals, and 6.9% (21 enterprises) employ over 500 individuals. Regarding the ownership structure, 24.2% (74 enterprises) are state-owned and 75.8% (232 enterprises) are non-state-owned.

### 3.2. Measures

This study measures humble leadership using the scale developed by Owens et al. (2013), which defines humble leadership through behaviors such as accurately assessing oneself, valuing others’ contributions and strengths, and being open to feedback or new ideas. This scale includes nine items scored on a 5-point Likert scale, ranging from “strongly disagree” (1) to “strongly agree” (5). A sample item from the scale is “The enterprise’s management actively seeks feedback, even if it is critical.” The Cronbach’s alpha for this scale was 0.96, indicating high reliability.

This study uses the scale developed by Bart and Cullen (1987) to measure the organizational caring ethical climate, defined as a climate based on consideration for others. The scale includes seven items scored on a 5-point Likert scale, with responses ranging from “strongly disagree” (1) to “strongly agree” (5). An example item from the scale is “What is best for everyone in the company is the major consideration here.” In this study, the Cronbach’s alpha for the scale was 0.95, indicating high reliability.

This study assesses organizational green innovation performance using a scale developed by Chang and Chen (2013). Green innovation involves technological advancements that save energy, decrease environmental pollution, recycle resources, and manage environmental protection. The scale includes eight items scored on a 5-point Likert scale, ranging from “strongly disagree” (1) to “strongly agree” (5). An example item is, “The company chooses materials for product development or design that produce the least amount of pollution.” In this study, the scale’s Cronbach’s alpha was 0.96, indicating high reliability.

This study measures the organizational structure using a scale developed by Z. Su et al. (2011). The organizational structure is defined as the formal plan of administrative systems and roles that manage resource flows and work activities (Olson et al., 1995). This scale comprises five items rated on a 5-point Likert scale. An example of an item on this scale is, “Please rate the trends in enterprise actions over the past three years: 1 = We prefer tight control of funds and operations through sophisticated control and information systems, 5 = We prefer loose, informal control and rely on informal relationships.” A higher score indicates a more organic organizational structure, while a lower score indicates a more mechanistic structure. In this study, the Cronbach’s alpha for the scale was 0.93, indicating high reliability.

This study uses the scale developed by Tan and Peng (2003) to measure unabsorbed organizational resource slack, which refers to resources that are currently not in use and are readily available for redeployment within the organization (Tan & Peng, 2003; Vanacker et al., 2017). The scale comprises three items scored on a 5-point Likert scale, ranging from “strongly disagree” (1) to “strongly agree” (5). A sample item from this scale is “The enterprise’s retained earnings have been sufficient for market expansion.” In this study, the Cronbach’s alpha for this scale was 0.91, indicating high reliability.

### 3.3. Data Analysis

First, the authors test the common method bias by examining the fit indices of measurement models, including a five-factor model and a one-factor model. Next, they examine the validity of the questionnaire through confirmatory factor analysis (CFA). Specifically, they verify convergent validity by calculating the average variance extracted (AVE) and composite reliability (CR) values. Additionally, they verify discriminant validity by comparing the square root of the AVE value with the correlation coefficient among the variables. Subsequently, the authors conduct a correlation analysis to provide initial evidence for the hypotheses. Finally, they perform hierarchical regression to test the hypotheses.

## 4. Results

### 4.1. Common Method Bias

To test for the presence of a common method bias, we conducted a confirmatory factor analysis (CFA) using the single-factor model approach. Specifically, we compared the fit indices of a one-factor model, in which all items were loaded on a single latent factor, to those of the proposed multi-factor model. If the common method bias is substantial, the single-factor model would exhibit a relatively good model fit, and the difference in the fit indices between the single-factor model and the hypothesized multi-factor model would not be significant. Conversely, if common method bias is not a major concern, the single-factor model would show a poor fit, and the hypothesized model would demonstrate a significantly better fit than the single-factor model (Podsakoff et al., 2003).

To detect the presence of common method bias, a CFA was conducted on the measures of humble leadership, an organizational caring ethical climate, green innovation performance, organizational structure, and unabsorbed organizational resource slack. This compared the fit indices of the hypothesized five-factor model with those of a one-factor model, which combined all five factors into one. A Chi-square to degrees of freedom ratio (χ^2^/df), Root Mean Square Error of Approximation (RMSEA), Comparative Fit Index (CFI), and Normed Fit Index (NFI) are commonly used fit indices in confirmatory factor analysis (CFA) to assess the overall model fit. A χ^2^/df value less than three generally indicates an acceptable fit, with values below two suggesting a good fit. For RMSEA, values below 0.08 indicate an acceptable fit, and values under 0.05 suggest a close fit. CFI and NFI values greater than 0.90 are typically considered acceptable, while values above 0.95 reflect a good model fit (Hair et al., 1998). These indices are interpreted collectively to determine the adequacy of the model in representing the observed data. The results showed that the hypothesized five-factor model fit the data well, whereas the one-factor model did not (see Table 1). Consequently, common method bias is not severe in this study.

### 4.2. Convergent Validity

This study confirmed the convergent validity by calculating the AVE and CR values. The widely accepted standard for good convergent validity dictates that the AVE value must exceed 0.5 and the CR value must exceed 0.7. As shown in Table 2, the AVE and CR values for all variables meet these criteria, indicating high convergent validity.

### 4.3. Discriminant Validity

This study confirmed discriminant validity by comparing the square root of the AVE for each main variable with its correlation coefficients with other main variables. Fornell and Larcker (1981) state that when the square root of the AVE for each main variable exceeds its correlation coefficients, the measurement model demonstrates good discriminant validity. As shown in Table 3, the measurement model in this study meets this criterion, indicating good discriminant validity among the variables.

### 4.4. Correlation Analysis

Table 3 shows that humble leadership significantly and positively correlates with both an organizational caring ethical climate (r = 0.288, *p* < 0.01) and green innovation performance (r = 0.418, *p* < 0.01). Additionally, there is a significant positive correlation between an organizational caring ethical climate and green innovation performance (r = 0.434, *p* < 0.01). The data also reveal a significant positive relationship between the organizational structure and green innovation performance (r = 0.438, *p* < 0.01). Furthermore, unabsorbed resource slack has a significant positive correlation with green innovation performance (r = 0.436, *p* < 0.01).

### 4.5. Test of Mediation Effect

Table 4 shows that humble leadership positively and significantly affects green innovation performance in enterprise management when controlling for variables such as the enterprise scale and ownership (B = 0.413, *p* < 0.01, Model 1), thus validating H1. In Model 1, the regression coefficient of humble leadership is 0.413, indicating a moderate positive effect of humble leadership on organizational green innovation performance. Additionally, the table indicates that, with sample feature variables controlled, humble leadership positively and significantly affects the organizational caring ethical climate (B = 0.292, *p* < 0.01), thus supporting H2. In Model 2, the regression coefficient of humble leadership is 0.292, suggesting a moderate positive influence on the caring ethical climate within the organization. In Model 3, the coefficients for humble leadership and a caring ethical climate are 0.317 and 0.328, respectively, and both are statistically significant. This suggests that both humble leadership and a caring ethical climate have moderate positive effects on organizational green innovation performance. Moreover, the simultaneous significance of these two variables provides preliminary evidence supporting the mediating role of a caring ethical climate.

As shown in Table 4, we tested Hypothesis 3 using Model 4 of Hayes’ PROCESS Macro, which is designed to assess simple mediation effects. Specifically, we examined whether an organizational caring ethical climate mediates the relationship between humble leadership and green innovation performance. We applied a bootstrapping procedure with 5000 resamples to estimate the indirect effect. The 95% bias-corrected bootstrap confidence interval for the indirect effect ranged from 0.056 to 0.1438 and did not include zero, indicating that the mediating effect is statistically significant. Therefore, Hypothesis 3 is supported.

### 4.6. Test of Moderation Effect

Table 5 shows that the interaction term (organizational caring ethical climate × organizational structure) positively and significantly affects green innovation performance when controlling for variables such as the enterprise scale and ownership (B = 0.211, *p* < 0.01). This finding validates H4. This result indicates that the organizational structure significantly and positively moderates the positive relationship between humble leadership and organizational green innovation performance, with a moderate effect size.

Table 5 shows that, when controlling for scale and ownership, the interaction term (organizational caring ethical climate × unabsorbed organizational resource slack) significantly and positively affects green innovation performance (B = 0.212, *p* < 0.01). This finding supports H5. This result indicates that unabsorbed slack resources significantly and positively moderate the positive relationship between humble leadership and organizational green innovation performance, with a moderate effect size.

**Figure 2:**
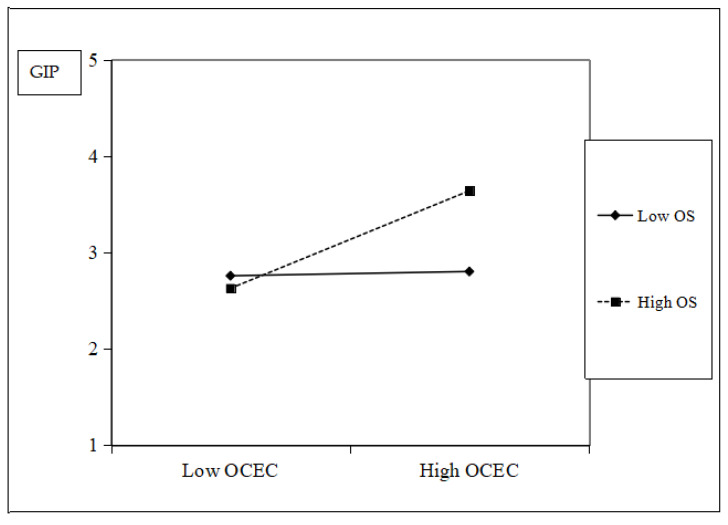
Moderating effect of organizational structure.

**Figure 3:**
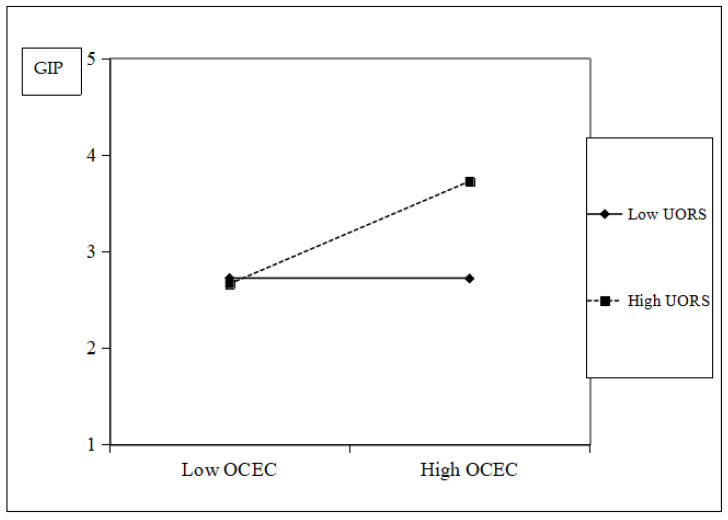
Moderating effect of unabsorbed organizational resource slack.

A simple slope plot was generated to illustrate the interaction effect. As Figure 2 shows, when the organizational structure is more organic (high OS), the positive relationship between an organizational caring ethical climate and organizational green innovation performance strengthens. Conversely, when the organizational structure is more mechanistic (low OS), this positive relationship weakens. Similarly, Figure 3 indicates that when unabsorbed organizational resource slack is high (high UORS), the positive relationship between an organizational caring ethical climate and organizational green innovation performance strengthens. However, when unabsorbed organizational resource slack is low (low UORS), this positive relationship weakens.

## 5. Discussion and Conclusions

### 5.1. Summary of Research Findings

This study reveals that humble leadership in enterprise management positively affects the green innovation performance of Chinese manufacturing enterprises. Additionally, an organizational caring ethical climate mediates this positive effect. Furthermore, the organizational structure and organizational resource slack serve as moderators in this relationship. Specifically, when the organizational structure is more organic and when unabsorbed organizational resource slack is high, the positive relationship between the organizational caring ethical climate and green innovation performance strengthens.

The theoretical mechanism behind the verified model demonstrates that followers adopt humble leaders’ positive behaviors, such as appreciating others’ contributions and being open to advice. These behaviors positively affect the organizational performance and demonstrate high consideration for the organization. Consequently, humble leadership contributes to a caring ethical climate within the organization, where there is mutual concern among members and the organization itself. Members perceive this kindness and good treatment, which motivates them to reciprocate with positive actions, such as enhancing the green innovation performance. Additionally, the organizational structure and organizational resource slack serve as promoting factors for green innovation performance, representing the ability and opportunity factors, respectively, in determining this performance. These two factors complement the motivation factor, which is the organizational caring ethical climate. Thus, the organizational structure and organizational resource slack both positively moderate the effect of the organizational caring ethical climate on green innovation performance.

### 5.2. Theoretical Implications

First, this study validates and enriches social learning theory by demonstrating that the positive effect of humble leadership on an organization’s caring ethical climate stems from followers learning humble leaders’ positive behaviors, such as appreciating others’ contributions and being open to others’ advice. This phenomenon supports the core principle of social learning theory, which states that people learn behaviors from role models (Bandura & Walters, 1977). Additionally, this study extends the application of social learning theory to explore the relationship between humble leadership in enterprise management and an organizational caring ethical climate.

Second, this study confirms and extends the application of social exchange theory by demonstrating how an organizational caring ethical climate positively affects green innovation performance. The mechanism behind this effect is that a caring ethical climate leads employees to perceive kindness and good treatment from the organization, which motivates them to reciprocate with positive behaviors such as high work effort and innovative actions. This finding supports the core principle of social exchange theory, which suggests that the perception of organizational benevolence encourages employees to reciprocate positively (Alalsheikh et al., 2022) Additionally, this study broadens the scope of social exchange theory by exploring its relevance to the relationship between an organizational caring ethical climate and green innovation performance.

Third, this study validates and enriches the COR theory by demonstrating that a caring ethical climate in organizations positively affects green innovation performance because it provides employees with essential social and psychological resources, motivating them to actively engage in resource investment behaviors, such as innovation. This supports the core premise of COR theory that individuals with abundant initial resources are more inclined to invest in such behaviors (Spanouli & Hofmans, 2021). Additionally, this study extends the application of COR theory by exploring the relationship between a caring ethical climate and green innovation performance.

Fourth, this study confirms and extends the application of AMO theory, which posits that ability, motivation, and opportunity are complementary factors in determining performance (Armstrong & Baron, 2005). The positive moderation effect of the organizational structure and unabsorbed organizational resource slack in this study stems from their complementarity with the motivation factor—specifically, a caring ethical climate—in enhancing green innovation performance. This finding not only supports the foundational concept of AMO theory, but also broadens its scope by exploring the moderators that influence the relationship between a caring ethical climate and green innovation performance.

Fifth, this study enhances research on the relationship between humble leadership and organizational green innovation performance. Previous studies have examined the relationship between a CEO’s humble leadership (individual level) and organizational green innovation performance (Sun et al., 2021); however, scarce research has focused on the relationship at the group level, specifically, the humble leadership of enterprise management. This study addresses this gap by having top managers assess both variables and further explores their relationship. Additionally, previous research has not clarified the mechanism by which humble leadership affects organizational green innovation performance (Sun et al., 2021). This study addresses this by examining the mediating role of the organizational caring ethical climate in the relationship between humble leadership and organizational green innovation performance.

Moreover, this study enhances research on the moderators that affect the relationship between an organization’s caring ethical climate and its green innovation performance. Previous studies have primarily examined how support for innovation, performance evaluation, and work autonomy moderate the relationship between an ethical climate and innovation (Kwon Choi et al., 2013; Santiago-Torner, 2023). However, few studies have explored the moderating roles of the organizational structure and resource slack. This study addresses this gap by examining how the organizational structure and resource slack affect the relationship between a caring ethical climate and green innovation performance.

Finally, this study compares its findings with prior empirical research and highlights the differences. First, some of the results align with those of Sun et al. (2021), specifically the positive impact of humble leadership on organizational green innovation performance. However, unlike Sun et al. (2021), the present study introduces new mediating and moderating variables into this relationship. Furthermore, the studies by Kelemen et al. (2023), Kwon Choi et al. (2013), and Andersson et al. (2020) suggest that a caring ethical climate may mediate the relationship between humble leadership and green innovation performance. This is consistent with the core findings of the present research; however, in contrast to these studies, this paper provides direct empirical evidence supporting this mediating role. Lastly, previous research has shown that the organizational structure and unabsorbed resource slack significantly influence the innovation performance (Dedahanov et al., 2017; Vanacker et al., 2017), which is consistent with this study’s findings. Nevertheless, this study distinguishes itself by introducing an interaction mechanism to examine these effects.

### 5.3. Practical Implications

First, this study finds that humble leadership can enhance the green innovation performance of organizations. Consequently, leaders in Chinese manufacturing enterprises should demonstrate a genuine desire for self-awareness, recognize and value the contributions and strengths of others, and remain open to feedback and new ideas to improve their enterprise’s green innovation performance. Additionally, these enterprises should offer sufficient leadership training to ensure their leaders are well-equipped with the necessary skills.

Moreover, this research reveals that leadership influences the green innovation performance of organizations significantly. To motivate leaders in enterprises to improve their leadership, organizations should listen to followers’ opinions about their leaders and seek their advice for leadership improvements. Organizations can incorporate followers’ remarks into the performance evaluation system of managers. In this way, managers in organizations can put more emphasis on followers’ feeling and improve their leadership according to the followers’ feedback.

Second, this study finds that an organizational caring ethical climate can enhance the green innovation performance. To achieve a better green innovation performance, Chinese manufacturing enterprises should develop an organizational culture that emphasizes employees’ common interests. Enterprises should encourage organizational citizenship behavior and reward it within their performance management and rewarding systems. Additionally, leaders should serve as role models by showing a high consideration for others.

Finally, this study identifies the organic organizational structure and organizational resource slack as two key factors that promote the green innovation performance in organizations. Therefore, to enhance green innovation, Chinese manufacturing enterprises should adopt a more organic organizational structure, promote open communication among employees, support the development of diverse skills, and empower employees to explore new ideas. Additionally, they should establish strong cooperation with stakeholders, such as banks and suppliers, because these stakeholders are crucial in providing support and resources for enterprise development.

### 5.4. Limitation and Future Research

First, all respondents in this study were recruited from manufacturing enterprises in Zhejiang province, raising concerns about whether they accurately represent manufacturing enterprises across China. Therefore, future research should validate the proposed model of this study using a more diverse sample.

Second, the mechanism by which humble leadership affects green innovation performance is relatively complex. There are likely other important mediators or moderators involved in this mechanism. Future research can further explore these related mechanisms.

Third, this study employed a cross-sectional design. From a rigorous perspective, this design does not suffice to prove a causal relationship. Therefore, future research should be longitudinal to validate the proposed model of this study.

Finally, the information source of this study is relatively single. The information of every enterprise is gathered from one manager in its top management team. This information gathering method increases the risk of common method bias. For future research, the authors advise that researchers should gather information from various sources.

## Figures and Tables

**Table 1:** Confirmatory factor analysis.

Model	χ^2^	df	χ^2^/df	RMSEA	CFI	NFI
Five-factor model (HL, OCEC, GIP, OS, UORS)	793.029	454	1.747	0.049	0.968	0.928
One-factor model (HL + OCEC + GIP + OS + UORS)	6845.166	464	14.753	0.212	0.394	0.379

Note: HL = Humble leadership, OCEC = Organizational caring ethical climate, GIP = Green innovation performance, OS = Organizational structure, UORS = Unabsorbed organizational slack.

**Table 2:** Convergent validity.

Convergent Validity		
Variable	AVE	CR
HL	0.748	0.964
OCEC	0.765	0.958
GIP	0.767	0.963
OS	0.755	0.939
UORS	0.798	0.922

**Table 3:** Correlation analysis.

	Mean	SD	Ownership	Scale	HL	OCEC	GIP	OS	UORS
Ownership	1.758	0.429	1						
Scale	2.431	0.740	0.061	1					
HL	3.213	1.048	0.013	−0.071	(0.865)				
OCEC	3.385	1.084	0.056	−0.094	0.288 **	(0.875)			
GIP	3.069	1.039	0.020	−0.049	0.418 **	0.434 **	(0.893)		
OS	3.364	1.060	0.100	−0.060	0.308 **	0.329 **	0.438 **	(0.876)	
UORS	3.261	1.161	0.075	−0.061	0.339 **	0.326 **	0.436 **	0.347 **	(0.869)

** *p* < 0.01; the square root of AVE is provided in parentheses.

**Table 4:** Result of mediation effect.

	GIP (Model 1)	OCEC (Model 2)	GIP (Model 3)
Constant	1.745 ** (5.248)	2.469 ** (6.779)	0.936 (2.805)
HL	0.413 ** (7.951)	0.292 ** (5.131)	0.317 ** (6.267)
OCEC			0.328 ** (6.673)
	306	306	306
*R* ^2^	0.176	0.092	0.282
**Control variables: scale, ownership**			
Direct effect	effect	lower limit	upper limit
HL → GIP	0.3172	0.2176	0.4168
Indirect effect	effect	lower limit	upper limit
HL → OCEC → GIP	0.0956	0.056	0.1438
**Control variables: scale, ownership**			

** *p* < 0.01; t-value in parentheses, beta values are shown above the parentheses.

**Table 5:** Result of moderation effect.

	Dependent Variable: GIP			
	*B*	*t*	*p*	*β*
constant	2.956 **	12.777	0.000	-
OS	0.167 **	3.499	0.001	0.17
UORS	0.204 **	4.623	0.000	0.228
OCEC	0.244 **	5.525	0.000	0.255
OCEC × UORS	0.212 **	5.625	0.000	0.260
OCEC × OS	0.211 **	4.347	0.000	0.204
**Control variable: scale ownership**				
n	306			
*R* ^2^	0.470			

** *p* < 0.01.

## Data Availability

The raw data supporting the conclusions of this study will be made available by the authors upon request.

## Abbreviations

HL = Humble leadership; OCEC = Organizational caring ethical climate; GIP = Green innovation performance; OS = Organizational structure; UORS = Unabsorbed organizational slack.
